# A Simple Method for In-Field Sex Determination of the Multicolored Asian Lady Beetle *Harmonia axyridis*

**DOI:** 10.1673/031.007.1001

**Published:** 2007-02-19

**Authors:** B. P. McCornack, R. L. Koch, D. W. Ragsdale

**Affiliations:** Department of Entomology, 219 Hodson Hall 1980 Folwell Avenue, University of Minnesota, St. Paul, Minnesota, 55108, USA

**Keywords:** *Harmonia axyridis*, sex determination, sexing, biological control, invasive species, temperature

## Abstract

The multicolored Asian lady beetle, *Harmonia axyridis* (Pallas) (Coleoptera: Coccinellidae), has become a popular study organism due to its promise as a biological control agent and its potential adverse, non-target impacts. Behavioral and ecological research on *H. axyridis*, including examinations of its impacts, could benefit from non-destructive or non-disruptive sexing techniques for this coccinellid. External morphological characters were evaluated for *H. axyridis* (*succinea* color form) sex determination in laboratory and field studies. The shape of the distal margin of the fifth visible abdominal sternite accurately predicted *H. axyridis* sex for all beetles examined. Males consistently had a concave distal margin, while females had a convex distal margin. In addition, pigmentation of the labrum and prosternum were both significantly associated with *H. axyridis* sex; males had light pigmentation and females had dark pigmentation. Labrum and prosternum pigmentation increased from light to dark with decreasing rearing temperature and increasing time after adult eclosion for females. Male pigmentation was only affected by a decrease in rearing temperature. Validation through in-field collections indicated that these predictors were accurate. However, labrum pigmentation is a more desirable character to use to determine sex, because it is more accurate and easily accessible. Therefore, we recommend using labrum pigmentation for in-field sex determination of *H. axyridis*. Implications of this diagnostic technique for applied and basic research on this natural enemy are discussed.

## Introduction

The multicolored Asian lady beetle, *Harmonia axyridis* (Pallas) (Coleoptera: Coccinellidae), is among the most studied aphidophagous predators, comprising 44% of studies on aphidophagous coccinellids published in 2004 (Sloggett 2005). This predator is native to eastern Asia, but has recently invaded North America, Europe, and South America (Koch 2003). Current populations of *H. axyridis* in these new regions likely resulted from intentional releases as a classical biological control agent (Gordon 1985); however, the possibility exists that it was accidentally introduced (Day et al. 1994). The popularity of *H. axyridis* as a study organism stems from its promise as a biological control agent and its potential adverse, non-target impacts. For example, *H. axyridis* is contributing to biological control of pest insects in a variety of systems (Koch 2003). However, *H. axyridis* is a threat to native species (Michaud 2002, Cottrell 2004, Koch et al. 2004b), becoming a pest of fruit production (Koch et al. 2004a), and can occasionally be a nuisance to home owners (Huelsman et al. 2002).

Behavioral and ecological research on *H. axyridis*, including examination of its positive and negative impacts, could benefit from in-field techniques for sexing this coccinellid. As mentioned by Majerus (1994), the sex of coccinellid adults can be easily determined through dissection, but more efficient techniques for sexing live adults are necessary. Non-destructive sex determination in coccinellids is generally difficult, with no characters applicable across the taxon (Majerus 1994; Hodek and Honek 1996). Despite the lack of all encompassing characters for sex determination, sexual dimorphism does appear to exist within most species. For many species, males are smaller with lighter pigmentation on the anterior portion of the head and slightly longer antennae (Hodek and Honek 1996). Of 24 British coccinellid species examined, 22 showed differences in the abdominal sternites among the sexes, but no difference was consistent across species (Majerus 1994). For *H. axyridis* in particular, adult females are generally larger than males, but the distributions of the size indices overlap (e.g., Hukusima and Ohwaki 1972; El-Sebaey and El-Gantiry 1999), so this is a less useful character. In addition, the last two visible abdominal sternites are sexually dimorphic and have been used to identify *H. axyridis* sex (Riddick and Schaefer 2005). However, this character is not readily visible on live field specimens unless they are manipulated and magnified.

The objectives of this project were to identify and quantitatively validate the accuracy of external characters of *H. axyridis* adults for rapid in-field sex determination. In particular, the robustness of these characters was examined across a range of conditions (i.e., temperature and diet) for laboratory-reared individuals and multiple temporal and spatial field collections of individuals.

## Materials and Methods

### Laboratory studies

*H. axyridis* adults were collected from an overwintering aggregation in an unheated building at University of Minnesota Outreach Research and Education (UMore) Park at Rosemount, MN on 7 October 2004. Following collection, beetles were held in 1.96 L plastic dishes with ∼200 beetles per dish, and maintained at 10 ± 1°C and a photoperiod of 16:8 (L:D). Prior to experimentation, the dishes containing beetles were warmed to 25 ± 1°C with a photoperiod of 16:8 (L:D), and the beetles were allowed to mate for 7 days. Beetles were provided an *ad libitum* supply of live soybean aphids, *Aphis glycines* Matsumura (Hemiptera: Aphididae), on excised soybean leaves (*Glycine max* (L.) Merrill) and water-soaked 9-cm^3^ cubes of Oasis floral foam (Smithers-Oasis Company, www.smithersoasis.com). After the mating period, adult females were maintained individually in plastic Petri dishes (60 × 15 mm) supplied with excised soybean leaves infested with soybean aphid and water-soaked, 1-cm^3^ foam cubes. Petri dishes containing females were checked daily for oviposition. If eggs were present, females were transferred to new petri dishes (60 × 15 mm) provisioned with soybean leaves, aphids and water. After egg hatch and dispersal of larvae from egg clusters (i.e., ∼1 day post hatching), first instars were individually placed into plastic Petri dishes (60 × 15 mm) and reared to adults under experimental conditions.

**Figure 1.**
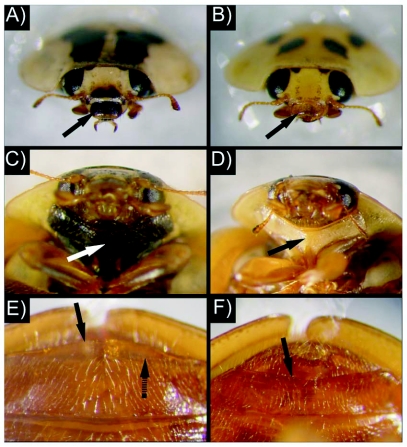
Labrum pigmentation (A—dark, B—light), protsternum pigmentation (C—dark, D—light), and the distal margin of 5th visible abdominal sternite (E—convex, F—concave) for *Harmonia axyridis* adults (A, C, E: female; B, D, F:male). Solid arrows (black and white) denote location of morphological characters used to predict sex. Note, the convex portion of the fifth visible abdominal sternite (dashed arrow) may appear transparent and could result in incorrect sex determinations.

The experimental conditions consisted of a three-factor factorial combination of temperature, diet, and time after adult eclosion. There were four temperatures (15, 20, 25, and 30°C), two diets (soybean aphids and pulverized, lyophilized honey bee pupae (*Apis mellifera* L.) (Okada and Matsuka 1973), and days after adult eclosion (1, 3, and 7 days). Individuals were reared in environmental growth chambers (Percival Scientific, www.percival-scientific.com) and were organized in a randomized complete block design. Each temperature was replicated twice starting with 45 *H. axyridis* for aphid diet and 30 for bee pupae diet within each replicate. After the specified time after adult eclosioin, *H. axyridis* labrum and prosternum pigmentation was recorded (without magnification) (i.e., light versus dark), and shape of the distal margin of the fifth visible abdominal sternite (i.e., concave vs convex) using a stereomicroscope (Figure 1). In a relatively small number of individuals (i.e., 0.07%), labrum or prosternum pigmentation was intermediate between light and dark. These individuals were grouped dark if at least 50% of the structure (i.e., labrum or prosternum) was darkly pigmented. At 7 days after eclosion, all individuals were dissected and sex was determined by the presence or absence of an aedeagus.

### Field studies

#### Seasonal and geographic variability

On 7 October 2004, *H. axyridis* adults were collected at UMore Park from an overwintering aggregation (fall collection 2004). From 18 May to 6 June 2005, adults were collected from buckthorn, *Rhamnus cathartica* L., at various locations throughout southern Minnesota (spring collection 2005; Table 3). On 27 July and 19 August 2005, adults were collected from soybean at UMore Park (mid-summer and late-summer 2005, respectively; Table 3). On 2 November 2005, adults were collected at UMore Park from the same overwintering site as in 2004 (fall collection 2005; Table 3). In addition, *H. axyridis* were obtained from different geographic locations. Adults were collected on 26 to 29 August 2005 from willow, *Salix* sp., at the Appalachian Fruit Research Station, Bardane, West Virginia. On 15 September 2005, adults were collected from Arlington Research Station, University of Wisconsin, Arlington, WI. *Harmonia axyridis* from both geographic locations were shipped overnight to the University of Minnesota, St. Paul, MN. Adults were then examined for labrum and prosternum pigmentation, shape of the distal margin of the fifth abdominal sternite, and dissected for presence or absence of an aedeagus as described previously.

### Days after eclosion

In a separate experiment, the effect of time after eclosion on labrum and prosternum pigmentation and shape of the distal margin of the fifth visible abdominal sternite was examined for newly emerged adults from a sample of field-collected *H. axyridis* pupae. On 19 August 2005, pupae were collected at UMore Park from soybean. Pupae were brought back to the lab and were maintained at 25 ± 1°C, 16:8 (L:D) in Petri dishes (60 × 15 mm). After eclosion, adults were supplied *ad libitum* with soybean leaves, aphids and water as described previously. Labrum and prosternum pigmentation, and shape of the distal margin of the fifth abdominal sternite were recorded at 0.3, 3, 7, 14, 21, and 35 days after adult eclosion. At 35 days after eclosion, all individuals were dissected and sex was determined by the presence or absence of an aedeagus as described previously.

### Statistical analysis

Least squares regression is limited in handling categorical variables with binary responses. Therefore, generalized linear models were used to analyze all data. Where appropriate, data were fit using simple and multiple logistic regression models and the logit (log odds ratio) link function was used to estimate various probabilities (Agresti 2002). The logit is the natural logarithm (ln) of odds of a successful response (e.g., adult female having a dark labrum or prosternum). Odds is defined as the probability of success over probability of failure. Therefore, an odds ratio is defined as the ratio between two odds. For example, when an odds ratio is significantly greater than 1 and less than infinity, individuals in one group are more likely to have a success than individuals from another group. For categorical (e.g., labrum and prosternum pigmentation) and continuous (e.g., temperature and time) predictors, the exponentiated slope estimate, β, for each predictor from the logistic regression model is simply the odds ratio, or the ratio of two odds. For example, the relationship between labrum pigmentation and *H. axyridis* sex can be interpreted by using the concept of odds ratio. The probability of success increases multiplicatively by the value of β for a 1 unit increase in the explanatory variable (e.g., temperature, time, diet, pigmentation).

For each study, maximum likelihoods (MLs) for intercept and slope parameters were estimated using the iterative, Newton-Raphson method for generalized linear models (R Project for Statistical Computing, Version 2.3.1). For model fitting, the most complex model was used, which included all main effects and interaction terms. Next, backward elimination was used and an appropriate logit model was selected using the likelihood-ratio statistic (i.e., differences in residual deviances between a fitted model and simpler model). Terms with the largest *P*-values were systematically removed until any further deletions yielded a significantly poorer fit (Agresti 2002). The Pearson's chi-squared statistic (χ^2^) and likelihood ratios (*G*^2^, saturated model deviance minus the residual deviance from nested model) were used to test the goodness-of-fit (i.e., H0: model fits data well) of each model. In addition, individual parameter estimates within each final model were tested (i.e., H_o_ : β = 0) using the Wald statistic (Agresti 2002).

For the laboratory study, temperature, diet, and days after eclosion were included as predictors of labrum pigmentation, prosternum pigmentation, or sternite shape. Therefore, logistic regression was used to investigate whether the above predictors were associated or conditionally independent of the odds of an adult having light or dark pigmentation on the labrum or prosternum. Categorical predictors that were not binary (e.g., temperature and time after eclosion) were treated as continuous variables in the logistic regression models. In addition, labrum pigmentation, prosternum pigmentation, or sternite shape were used as separate predictors of *H. axyridis* actual sex for the laboratory study and both field studies.

**Table 1.**
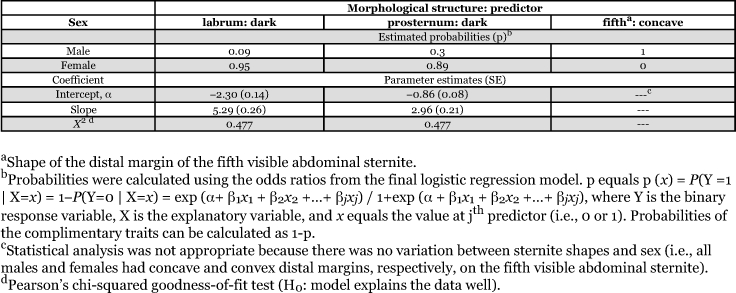
Association between morphological characters (i.e., labrum and prosternum pigmentation and sternite patterns) and *H. axyridis* sex (determined by dissection) across diets, temperatures, and days post adult emergence.

## Results

### Laboratory study

Eclosion of beetles from bee pupae and aphid diets was 40 and 62% at 15°C, 75 and 77% at 20°C, 77 and 61% at 25°C, and 57 and 67% at 30°C, respectively. Across all replications, temperatures, diets, and days after eclosion, females consistently had a convex distal margin for the fifth abdominal sternite. Conversely, males consistently exhibited a concave distal margin (Table 1). Therefore, a statistical model for predicting sex based on the fifth visible abdominal sternite shape was not appropriate, because this character was always associated with sex (i.e., there was no variation). Labrum pigmentation was significantly associated with *H. axyridis* sex across all diets, temperatures, and days after adult eclosion (Table 1). The likelihood-ratio test statistic comparing the fitted model (i.e., main effect of labrum pigmentation) to the independence model (i.e., no effect term) shows strong evidence for an association (*G*^2^ = 898.2; df = 1; *P* < 0.001). In addition, Pearson's χ^2^ statistic showed the model fit the data well (χ^2^ = 1041; df = 1039; *P* = 0.477). The Wald statistic (i.e., z^2^ = (β/SE)^2^ = 399.5; df = 1; *P* < 0.001) also showed strong evidence of an association between labrum pigmentation and sex (Table 1). The odds of success for adult *H. axyridis* having a dark labrum is 198.1 times greater for females than males; the Wald 95% confidence interval for this relationship is 197.5 to 198.6. Therefore, the estimated probability of a female having a dark labrum from the logistic regression is 0.952 (Table 1).

**Table 2:**
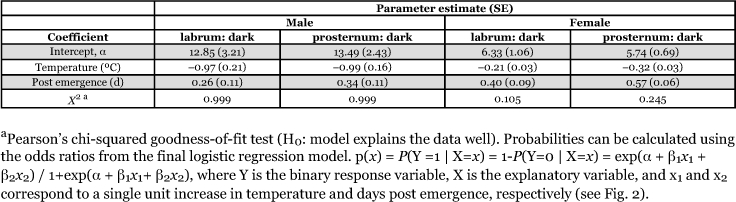
Associations between temperature, days post emergence, and pigmentation (labrum and prosternum) for *H. axyridis* males and females.

**Figure 2.**
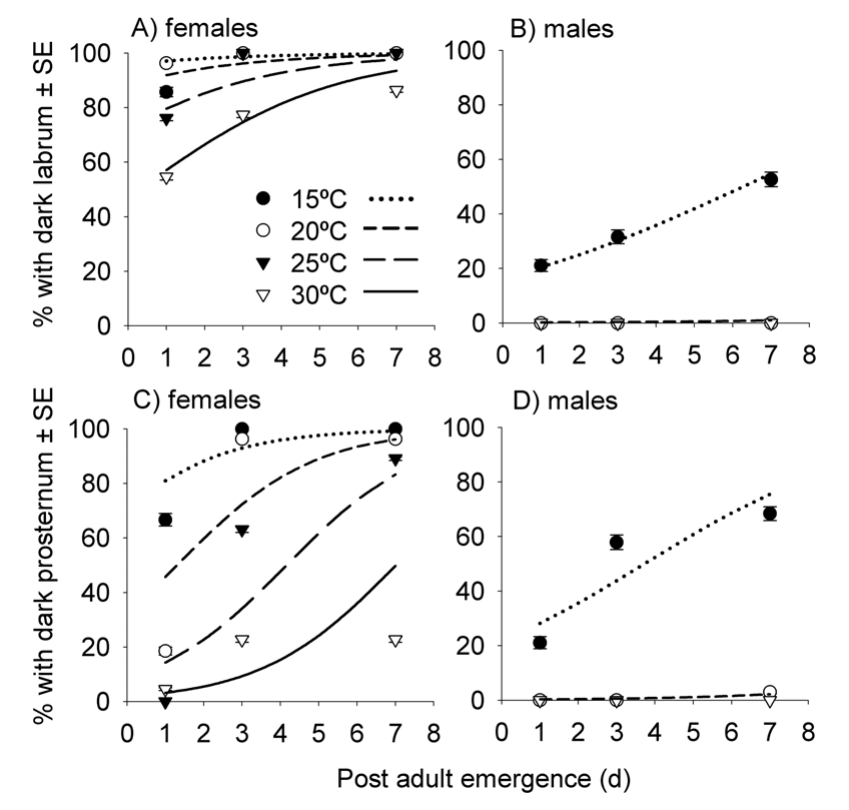
Effect of temperature and time post adult eclosion on labrum (A, B) and prosternum pigmentation (C, D) for laboratory reared *Harmonia axyridis* males (B, D) and females (A, C). Symbols represent observed data and lines represent predictions from logistic regression models (see Table 2 for parameter estimates).

Prosternum pigmentation was also significantly associated with *H. axyridis* sex across all diets, temperatures, and days after adult eclosion (Table 1). The likelihood-ratio test statistic comparing the fitted model (i.e., main effect of prosternum pigmentation) to the independence model (i.e., no effect term) showed strong evidence of an association (*G*^2^ = 313.0; df = 1; *P* < 0.001) and Pearson's Χ^2^ statistic showed the model fit the data well (Χ^2^ = 1041; df = 1039; *P* = 0.477). The Wald statistic (i.e., z^2^ = 200.9; df = 1; *P* < 0.001) also showed strong evidence of an association between prosternum pigmentation and sex (Table 1). However, the odds of success for adult *H. axyridis* having a dark prosternum was 19.3 times greater for females than males; the Wald 95% confidence interval for this relationship is 18.9 to 19.7. Therefore, the estimated probability of a female having a dark prosternum from the logistic regression was 0.891 (Table 1). Overall, predicting *H. axyridis* sex using labrum pigmentation was less variable than using prosternum pigmentation.

For males and females, separate logistic models with labrum or prosternum pigmentation as the response included only the main effects of temperature and days after eclosion (Table 2). Diet and replicate were conditionally independent of labrum or prosternum pigmentation. Therefore, the main effects (i.e., temperature and days after eclosion) were collapsed over replicate and diet. Thus, the estimated treatment effect is approximately the marginal odds ratios of temperature and days after eclosion on labrum or prosternum pigmentation.

**Table 3.**
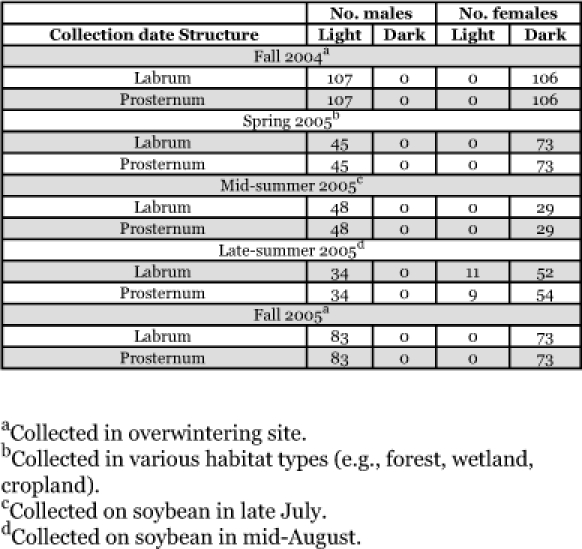
Labrum and prosternum pigmentation for field-collected *H. axyridis* at different times during the year in Minnesota.

For labrum pigmentation, the likelihood-ratio test statistic comparing the fitted model to the independence model showed strong evidence of an association for females (*G*^2^ = 66.7; df = 2; *P* < 0.001) and males (*G*^2^ = 98.78; df = 2; *P* < 0.001). Pearson's χ^2^ statistic showed the models fit the data well for female (χ^2^ = 509.9; df = 471; *P* = 0.105) and male (χ^2^ = 161.9; df = 564; *P* = 0.999) individuals. Wald statistics also showed strong evidence of an association between labrum pigmentation and rearing temperature for females (z^2^ = 29.70; df = 1; P < 0.001) and males (z^2^ = 21.25; df = 1; *P* < 0.001). In addition, there was a significant association between labrum pigmentation and days after eclosion for females (z^2^ = 21.28; df = 1; *P* < 0.001) and males (z^2^ = 5.27; df = 1; *P* = 0.02) (Table 2). Dark labrum pigmentation was negatively affected by temperature (i.e., -β) and positively affected by days after eclosion (i.e., + β) (Table 2). For females and males, odds of having a dark labrum decreased 80.7 and 38.1%, respectively, for every 1 unit increase in temperature (Table 2). Conversely, odds of adult *H. axyridis* having a dark labrum increased 48.9% for females and 29.3% for males for every 1 unit increase in days after eclosion(Table 2). In this study, all adults had the highest probability of a dark labrum at the lowest rearing temperatures (i.e., 15°C) and greatest days after eclosion (i.e., 7 days) (Figure 2).

Prosternum pigmentation also showed strong evidence of an association for females (*G*^2^ = 248.8; df = 2; *P* < 0.001) and males (*G*^2^ = 143.9; df = 2; *P* < 0.001) with the main effects of temperature and days after eclosion; data were collapsed across diet and replicate. Pearson's χ^2^ statistics showed the models fit the data well for female (χ^2^ = 491.9; df = 471; *P* = 0.245) and male (χ^2^ = 150.4; df = 564; *P* = 0.999) individuals. Wald statistics also showed strong evidence of an association between prosternum pigmentation and rearing temperature for females (z^2^ = 99.86; df = 1; *P* < 0.001) and males (z^2^ = 37.81; df = 1; *P* < 0.001). In addition, there was a significant association between prosternum pigmentation and days after eclosion for females (z^2^ = 85.92; df = 1; *P* < 0.001) and males (z^2^ = 9.13; df = 1; *P* = 0.003) (Table 2). Dark prosternum pigmentation was negatively affected by temperature and positively affected by days after eclosion (Table 2). For females and males, odds of having a dark prosternum decreased 72.3 and 37.4%, respectively, for every 1 unit increase in temperature (Table 2). Conversely, odds of adult *H. axyridis* having a dark prosternum increased 76.2% for females and 40.9% for males for every 1 unit increase in days after eclosion (Table 2). All adults had the highest probability of a dark prosternum at the lowest rearing temperatures (i.e., 15°C) and greatest days after eclosion (i.e., 7 days) (Figure 2).

### Field studies

#### Seasonal and geographic variability

In the field-collected validation, 100% of the males collected had light labrums and prosternums; however, variability in labrum and prosternum pigmentation was observed for late-summer collected females (Table 3). To test for the association between labrum or prosternum pigmentation and *H. axyridis* sex for beetles observed on 19 August, Fisher's exact test was used for count data having a small-sized distribution (i.e., cell counts < 5) (Agresti 2002). For labrum and prosternum pigmentation, the true odds ratios were significantly different from 1 (*P* < 0.001) and both characters had a Wald 95% confidence interval between 0.00 and 0.03, suggesting a strong association between labrum and prosternum pigmentation and sex. Furthermore, in field-collections of *H. axyridis* from West Virginia (129 adults) and Wisconsin (26 adults), 100% of the females that were collected had a dark labrum and prosternum and 100% of the males had a light labrum and prosternum.

**Figure 3.**
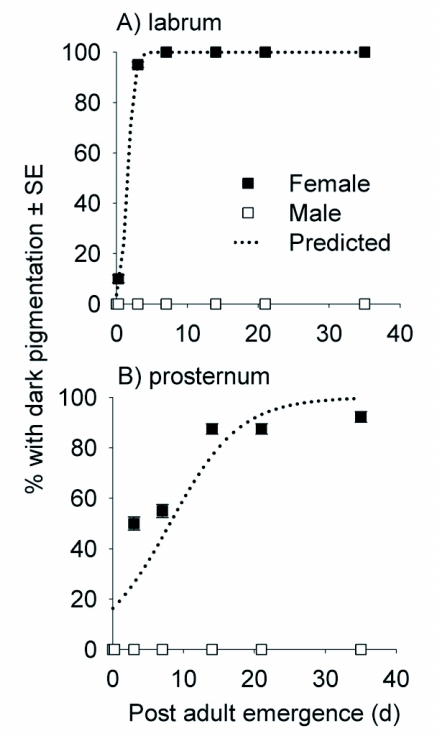
Relationship between labrum and prosternum pigmentation and days post adult eclosion for field-collected *Harmonia axyridis* pupae maintained at 25 ± 1°C in an environmental growth chamber. Symbols represent observed data and dotted lines represent predictions from logistic regression models (see Table 4 for parameter estimates). Since all males had light labrums and prosternums, predictions from the logistic regression model (dotted line) only pertain to female *H. axyridis*.

### Days after eclosion

For the 32 males that emerged from field-collected pupae, there was no variability in labrum or prosternum pigmentation as all males had light pigmentation. However, for females, labrum and prosternum pigmentation varied. Therefore, only the female data in the logistic regression model were included. For female labrum pigmentation, the likelihood-ratio test statistic comparing the fitted model to the independence model showed evidence of a strong association (*G*^2^ = 133.1; df = 1; *P* < 0.001), and Pearson's χ^2^ statistic showed the model fit the data well (χ^2^ = 54.5; df = 124; *P* = 0.999). From the logistic regression model, the odds of a female having a dark labrum increased 8.14 times for every 1 unit increase in days after eclosion; 100% of *H. axyridis* females had dark labrums by 7 days (Figure 3, Table 4). In addition, female prosternum pigmentation was associated with days after eclosion (*G*^2^ = 61.8; df = 1; *P* < 0.001) and Pearson's χ^2^ statistic showed the model fit the data well (χ^2^ = 112.9; df = 124; *P* = 0.754). Similarly, the odds of a female having a dark prosternum increased by 22.6% for every 1 unit increase in days after eclosion; only 92.3% of the females had a dark prosternum by the end of the 35 day study (Table 4; Figure 3). Conversely, males never had a dark labrum or prosternum at any time during the study (Figure 3).

**Table 4.**
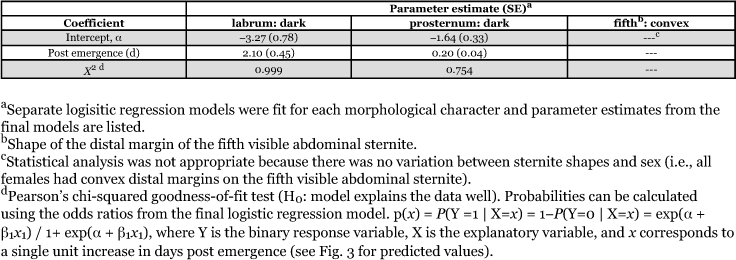
Associations between days post emergence and labrum and prosternum pigmentation for *H. axyridis* females emerged from field-collected pupae. For all males, there was no variability in labrum or prosternum pigmentation and only female data were included in the final logistic regression models.

## Discussion

Methods for determining the sex of coccinellids without dissection are necessary for ecological and genetic studies (Majerus 1994). Our results indicate that external morphological characters can be used to determine the sex of *H. axyridis* without killing the beetles. The shape of the distal margins of the last visible abdominal sternites has been used for sex determination for various coccinellids (Baungaard 1980; Majerus 1994; Hodek and Honek 1996), including *H. axyridis* (Riddick and Schaefer 2005). However, the error rates associated with this technique have not been quantified. The shape of the distal margin of the fifth visible abdominal sternite was found to accurately predict *H. axyridis* sex for all beetles examined. Males consistently had a concave distal margin, while females had a convex distal margin (Figure 1E-F). In contrast, Tsaganou et al. (2004) mentioned that the shape of the abdominal sternites was not a reliable predictor of *H. axyridis* sex. This discrepancy among studies may be due to the convex portion of the fifth visible abdominal sternite occasionally being transparent, thus appearing concave and resulting in true females being classified as males (Figure 1). At times it was necessary to use a fine pointed probe to identify the true distal margin of the abdominal sternite.

Since adult *H. axyridis* must be manipulated (i.e., handled and inverted) and magnified when using the shape of the fifth visible abdominal sternite for sex determination, other more readily-accessible morphological characters were sought for determining the sex of *H. axyridis*. Labrum and prosternum pigmentation were both significantly associated with *H. axyridis* sex. Of these two characters, labrum pigmentation is more desirable, due to its higher accuracy at predicting sex. In addition, *H. axyridis* can be sexed in the field using labrum pigmentation often without magnification or manipulation. If a hand lens is required, sex identification can be recorded with minimal disturbance to the adult unlike the use of sternal characters, which would always require adult disturbance and manipulation to view in the field. Rogers et al. (1971) also used differences in pigmentation of the anteromedian portion of the head to differentiate the sexes of *Propylea 14-punctata* (L.).

For a character such as labrum or prosternum pigmentation to be employed for sex determination its robustness to change under varying conditions must be evaluated. For instance, Grill and Moore (1998) found that diet can influence the coloration of *H. axyridis*. However, we found that labrum and prosternum pigmentation were conditionally independent of diet (i.e., live aphids vs the diet made from bee pupae). In addition, rearing temperature has also been shown to influence the coloration of *H. axyridis* elytral pattern (Sakai et al. 1974). Our results extend this tendency to other structures (i.e., labrum and prosternum), with darker labrum and prosternum pigmentation being associated with lower constant temperatures. In our study, time after adult eclosion also had a significant impact on labrum and prosternum pigmentation. For females, these characters became darker with increasing time after adult eclosion, whereas the labrum and prosternum of males remained light through time. For laboratory-reared or field-collected females, variability in labrum pigmentation only lasted for 3 days after adult eclosion (Figures 2, 3). This result comes as no surprise, since it is well known that sclerotization and pigmentation of adult insects increases with increasing time after adult eclosion (Chapman 1998). However, prior to our study the time required for this change to occur had not been determined for labrum and prosternum pigmentation of *H. axyridis*.

As validation for the use of labrum or prosternum color for sex determination, the temporal and spatial variability of these characters was examined as predictors of sex for field-collected *H. axyridis*. Among the additional locations (i.e., Wisconsin and West Virginia) from which *H. axyridis* were obtained, labrum and prosternum pigmentation proved accurate for predicting sex. Furthermore, for beetles collected throughout the year in Minnesota, these predictors maintained their accuracy, with the labrum and prosternum being light in all males and dark in all females; except in the late summer collection, when variability was observed among the females. The presence of females with light labrums in late summer was likely attributed to this collection date coinciding with the peak eclosion of adult *H. axyridis* from pupae in the field (B.P.M., unpublished data) and the associated variability in pigmentation of the characters of females shortly after eclosion (as mentioned above).

Intended uses for labrum pigmentation as a predictor of *H. axyridis* sex include both applied and basic research in the laboratory and field. For example, augmentative biological control programs utilizing *H. axyridis* could benefit from a more rapid method of sorting males from females when mass-rearing this predator. In addition, this readily usable technique for in-field sex determination could be used to improve studies on the population dynamics and behavior of this beneficial/pest insect. In such studies, *H. axyridis* sex could be determined without disturbing or removing beetles from their natural environment. Furthermore, this technique could provide for rapid and accurate screening of *H. axyridis* for long-term monitoring of female-biased sex ratios potentially resulting from infection with male-killing bacteria (Majerus et al. 1999; Nakamura et al. 2005). For instance, in studies on the population dynamics of most species, females are considered most important for population growth and are often the only sex counted in modeling exercises. In the field it can be difficult to determine sex ratios, so a sex ratio of 1:1 is often assumed (Galvan et al. 2005). However, the presence of male killing bacteria could alter sex ratios, potentially making these assumptions less valid. Thus, rather than assuming a sex ratio, the sex of individuals observed in field and laboratory studies should be determined to provide more accurate estimates.

Our studies included only the *succinea* color morph (i.e., orange to red elytra with or without black spots) of *H. axyridis*, since this has been the only color morph documented throughout North America (Chapin and Brou 1991; Tedders and Schaefer 1994; Dreistadt et al. 1995; Kidd et al. 1995; Hesler et al. 2001; Wise et al. 2001) and South America (Saini 2004; de Almeida and da Silva 2002) except in western Oregon where melanic morphs have been collected at low rates (1.4% of population) (LaMana and Miller 1996). Further work is required to determine effectiveness of these traits for predicting sex in the melanic color morphs of *H. axyridis* that are more common in Asia and Europe (Komai 1956; Majerus and Roy 2005).
